# Encéphalopathie de Gayet-Wernicke avec taux de thiamine normale: à propos d’un cas

**DOI:** 10.11604/pamj.2021.38.16.25888

**Published:** 2021-01-07

**Authors:** Assia El Berhoumi, Dalale Laoudiyi, Meriem Doumiri, Hayat Lhajoui, Mohamed Labied, Kamilia Chbani, Siham Salam, Lahcen Ouzidane

**Affiliations:** 1 Service de Radiologie Pédiatrique, Hôpital d´Enfants Abderrahim Harouchi, Centre Hospitalier Universitaire Ibn Rochd, Casablanca, Maroc

**Keywords:** Gayet-Wernicke, imagerie par résonance magnétique, thiamine, *case report*, Gayet-Wernicke, magnetic resonance imaging, thiamine, case report

## Abstract

Résumé

L'encéphalopathie de Gayet-Wernicke est une urgence neuropsychiatrique sur carence en thiamine (vitamine B1), secondaire à plusieurs facteurs. Nous rapportons un cas d'encéphalopathie de Gayet-Wernicke chez une patiente de 43 ans non éthylique, se présente pour trouble de conscience et diplopie, avec un taux de thiamine normal. La triade clinique classique et l'imagerie par résonance magnétique (IRM) jouent un rôle important, notamment dans le diagnostic des encéphalopathies de Wernicke non alcooliques même avec un taux de thiamine normal.

## Abstract (en)

Gayet-Wernicke encephalopathy is a neuropsychiatric emergency due to thiamine deficiency (vitamin B1), secondary to several factors. We here report a case of Gayet-Wernicke encephalopathy in a 43-year-old woman who didn’t consume alcoholic beverages, presenting with disorders of consciousness and diplopia with normal thiamine level. Classic triad of symptoms and Magnetic resonance imaging (MRI) played an important role, in particular, in the diagnosis of Gayet-Wernicke encephalopathy with normal thiamine levels in the absence of alcohol abuse.

## Introduction

L'encéphalopathie de Gayet-Wernicke est un syndrome neuropsychiatrique aigu et grave, initialement décrit en 1881 par Carl Wernicke [1]. C´est une maladie rare [2]. Ses symptômes regroupent la triade classique l'ataxie, l'ophtalmoplégie et la confusion [1]. Elle a généralement été très fréquemment évoquée dans le contexte de l'alcoolisme et des signes neurologiques, mais on la retrouve aujourd'hui dans divers tableaux cliniques [3]. Cependant, il est impératif de diagnostiquer la maladie et de commencer le traitement le plus rapidement possible car elle reste un diagnostic clinique qui peut être établi même avec des taux de thiamine sanguine normaux et avec une imagerie par résonance magnétique (IRM) ayant une sensibilité de 53% et une spécificité de 93% [3]. Nous avons eu un cas d´encéphalopathie de Wernicke après une durée prolongée de malnutrition et de vomissements.

## Patient et observation

**Figure 1:**
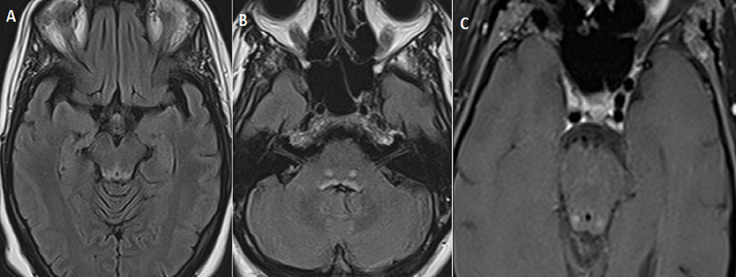
(avant traitement) A) séquence axiale FLAIR: hypersignal bilatéral et symétrique en périaqueducale des corps mamillaires et des colliculi; B) séquence axiale FLAIR: hypersignal bilatéral et symétrique des noyaux dorsaux du pont; C) séquence axiale T1 SE après injection de gadolinium: rehaussement nodulaire des colliculi

**Figure 2:**
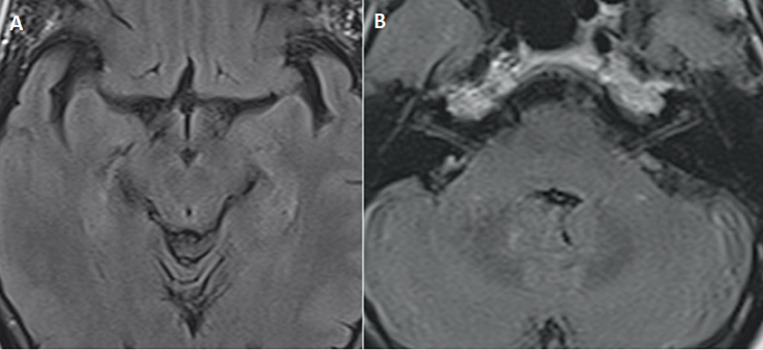
(IRM de contrôle après 4 mois) séquence axiale FLAIR: disparition totale des hypersignaux au niveau périacqueducal, des colliculi et des noyaux dorsaux du pont

Une patiente âgée de 43 ans, diabétique sous insuline, non alcoolique, admise dans notre structure, en février 2020, dans un tableau associant un trouble de conscience apyrétique à type de confusion, une diplopie binoculaire horizontale avec diminution de l´abduction à droit, des vomissements prolongés et une malnutrition sévère suite à une hospitalisation prolongée en réanimation. L'IRM cérébrale montrait des hypersignaux T2 et FLAIR bilatéraux et symétriques de la région périacqueducale, des tubercules cérébraux (colliculi), des corps mamillaires (Figure 1A) et des noyaux dorsaux du pont (Figure 1B), sans traduction sur la diffusion. Une prise de contraste nodulaire des tubercules cérébraux (Figure 1C), l´absence d´anomalie de signal des nerfs VI, des nerfs optiques, et des muscles oculomoteurs. Le diagnostic d'encéphalopathie de Gayet Wernicke était retenu. Le dosage de la vitamine B1 était normal à 125nmol/l (N=67-200). Elle a quand même bénéficié d'un traitement injectable dès la suspicion diagnostique, suivi d'un relais oral. L'évolution était favorable avec amélioration de la conscience au bout de 4 jours et disparition de la diplopie. Revue quatre mois plus tard, la patiente présentait un bon état général et une IRM cérébrale normale (Figure 2).

## Discussion

L'encéphalopathie de Gayet-Wernicke est une complication neuropsychiatrique aiguë et grave due à une carence en vitamine B1 (thiamine). C'est une maladie rare [1, 4]. Décrite pour la première fois en 1881 par Carl Wernicke chez un homme alcoolique et une femme présentant des vomissements incoercibles [2, 5]. Elle touche le plus souvent l´homme que la femme et la prévalence autoptique estimée à 0,8-2,8% est beaucoup plus élevée que celle observée avec les manifestations cliniques (0,04-0,13%) [2]. L'encéphalopathie de Gayet-Wernicke est le plus souvent observée lors d'un alcoolisme chronique si l'apport alimentaire de thiamine n´est pas adéquat, mais de manière plus générale dans un contexte de dénutrition sévère. Ainsi, les autres causes sont les vomissements gravidiques ou prolongés, l'alimentation parentérale prolongée, la grève de la faim, l'anorexie mentale, les chirurgies gastro-intestinales, les cancers et la chimiothérapie et plusieurs d´autres facteurs [6]. Chez notre patiente, les deux facteurs incriminés dans la survenue d'une encéphalopathie de Gayet-Wernicke étaient les vomissements incoercibles et la dénutrition sévère ayant duré un mois et demi, sans supplémentation vitaminique.

Le déficit en thiamine entraine des lésions cérébrales, en 2 à 3 semaines, de sévérité variable, allant des lésions hémorragiques pétéchiales et de l´œdème à l´atrophie et la destruction des neurones. Seuls 16,5% des patients présentent la triade classique: ataxie, ophtalmoplégie et confusion, alors qu'environ 19% des patients ne présentaient aucun des symptômes classiques. La confusion est le symptôme le plus commun dans environ 82% des cas, suivi par les troubles oculomoteurs dans 29%, l'ataxie de la marche dans 23% et la polyneuropathie dans 11%. Les tableaux cliniques atypiques comprennent la stupeur, l'hypotension, le dysfonctionnement vestibulaire sans perte auditive [3, 7]. Devant une suspicion d'encéphalopathie de Gayet-Wernicke, l'IRM constitue l'examen de référence avec une sensibilité de 53% et une spécificité de 93% [3]. Elle montre des hypersignaux en T2, FLAIR et diffusion, typiques par leur localisation et leur caractère symétrique et bilatéral, sans ou avec prise de contraste après injection de Gadolinium. Les lésions IRM impliquent classiquement le thalami médial, en particulier le long de la paroi du troisième ventricule (80% à 85%), les zones périaqueducales (59% à 65%), les corps mamillaires (38% à 45%), la plaque tectale (36% à 38%) et la moelle dorsale avec l'implication des noyaux hypoglosses (8%). D'autres sites moins courants sont le corps calleux, le noyau caudé, le noyau rouge, le noyau denté, le cervelet, l'hippocampe et les cortex cérébraux frontal et pariétal [3]. Une imagerie normale n'élimine pas le diagnostic.

La vitamine B1 est présente dans l'alimentation. Les besoins journaliers sont de l'ordre de 1,5mg/j [8]. L'absorption se fait au niveau de l´intestin grêle proximal. Après absorption intestinale, la thiamine est phosphorylée dans le foie pour donner le pyrophosphate de thiamine: c´est la forme active de la vitamine B1 et un coenzyme essentiel de plusieurs réactions biochimiques [8]. La vitamine B1 circule dans les globules blancs, globules rouges et plaquettes. Elle diffuse dans les organes les plus consommateurs, le cœur, le foie, le cerveau et les reins [8]. La thiamine est essentiellement transportée à travers la barrière hémato-encéphalique par des transporteurs (transport actif) et par un processus lent et passive (lorsque la concentration dépasse le seuil de transport actif) [6, 8]. On pourrait, par définition, s'attendre à un faible niveau de thiamine dans l'encéphalopathie de Wernicke. Néanmoins, les tests de thiamine ont des limites bien connues. Les taux sériques de thiamine sont une mauvaise mesure du statut de la thiamine [6, 9].

Plusieurs laboratoires en Australie utilisent désormais la chromatographie liquide à haute performance avec détection fluorescente pour mesurer le pyrophosphate de thiamine dans le sang [6]. On pense que ce test améliore la reproductibilité, mais il a également été constaté que le processus de normalisation de ses méthodes chromatographiques complexes présentait des faiblesses [6, 9]. En effet, bien que ces analyses donnent une estimation des taux de thiamine dans le sang entier, elles ne reflètent pas nécessairement les taux de thiamine intracérébrale [6]. Ce dosage ne représente que 0,8% des stocks de l´organisme en vitamine B1 [8]. Les taux normaux varient de 67 à 200nmol/l dans le sang global chez l'adulte [6]. Le taux de thiamine dosé au niveau du sang pouvant être normal, différenciant du taux réel de thiamine en intracérébral.

L'encéphalopathie de Wernicke ne peut être diagnostiquée simplement en mesurant le taux de thiamine circulant [6]. D´où la priorité de l´IRM cérébrale dans les investigations complémentaires. Comme cette entité pathologique est une urgence médicale de diagnostic pouvant être clinique même avec une IRM et des niveaux de thiamine sanguine normaux. Le traitement doit être précoce, dès la suspicion diagnostique sans être retardé par les dosages vitaminiques et devra être poursuivi jusqu'à l´amélioration clinique [5, 10]. Le pronostic de l'encéphalopathie de Gayet-Wernicke est variable. Il est favorable lorsque la maladie est diagnostiquée précocement avec un traitement adéquat et une rémission complète des symptômes, comme le cas chez notre patiente ayant normalisé son IRM de contrôle. Seulement 16% des patients insuffisamment traités se rétablissent complètement alors que d´autres développent le syndrome de Korsakoff. Le taux de mortalité est de 17-20% [10]. La prévention passe par une administration parentérale de vitamine B1 à tous les patients à risque [2].

## Conclusion

L'encéphalopathie de Gayet-Wernicke est une maladie rare et grave. Devant une situation à risque, la triade ophtalmoplégie, confusion mentale et ataxie est évocatrice. L'IRM constitue l'examen de référence et le dosage vitaminique se fera si nécessaire et possible. Un taux de thiamine normal n´élimine pas le diagnostic d´encéphalopathie de Gayet-Wernicke. Le traitement précoce permet d'avoir une évolution favorable et d'éviter l'installation d'un syndrome de Korsakoff.

## References

[1] Han JW, Lim S, Shin HS, Park HJ, Jung WJ, Kwon SY (2012). Two cases of Wernicke´s encephalopathy in young age patients receiving allogeneic hematopoietic stem cell transplantation. Yonsei Med J.

[2] Davies SB, Joshua FF, Zagami AS (2011). Wernicke´s encephalopathy in a non-alcoholic patient with a normal blood thiamine level. Med J Aust.

[3] Segal JB, Bouffard MA, Schlaug G (2016). Characteristic neuroimaging abnormalities of Korsakoff Syndrome. JAMA Neurology.

[4] Patel S, Topiwala K, Hudson L (2018). Wernicke's encephalopathy. Cureus.

[5] de Oliveira AM, Paulino MV, Vieira APF, McKinney AM, da Rocha AJ, Dos Santos GT (2019). Imaging patterns of toxic and metabolic brain disorders. Radiographics.

[6] Seci G, Sera A (2007). Wernicke's encephalopathy: new clinical settings and recent advances in diagnosis and management Lancet Neurol. Lancet Neurol.

[7] Thomson AD, Cook CCH, Touquet R, Henry JA, Royal College of Physicians-London (2002). The Royal College of Physicians report on alcohol: guidelines for managing Wernicke's encephalopathy in the accident and emergency department. Alcohol Alcohol.

[8] Quilliot D, Michot N, Brunaud L, Malgras A (2017). Déficit en vitamine B1: comment prévenir et traiter. Nutrition Clinique et Métabolisme.

[9] Welsh A, Rogers P, Clift F (2016). Nonalcoholic Wernicke's encephalopathy. CJEM.

[10] Kumar N (2010). Neurologic presentations of nutritional deficiencies. Neurol Clin.

